# Assessing Ghana’s eHealth workforce: implications for planning and training

**DOI:** 10.1186/s12960-018-0330-8

**Published:** 2018-11-27

**Authors:** Henry A. Ogoe, James A. Asamani, Harry Hochheiser, Gerald P. Douglas

**Affiliations:** 1 Department of Biomedical Informatics, University of Pittsburgh, 5607 Baum Boulevard, Pittsburgh, PA 15206 United States of America; 2 Department of Biomedical Engineering, University of Ghana, Legon, Ghana; 3 Human Resource Division, Ghana Health Service, Accra, Ghana; 4 Intelligent Systems Program, University of Pittsburgh, Pittsburgh, PA United States of America

**Keywords:** eHealth workforce, LMICs, Staffing needs, WISN, Workload, Ghana

## Abstract

**Background:**

eHealth—the proficient application of information and communication technology to support healthcare delivery—has been touted as one of the best solutions to address quality and accessibility challenges in healthcare. Although eHealth could be of more value to health systems in low- and middle-income countries (LMICs) where resources are limited, identification of a competent workforce which can develop and maintain eHealth systems is a key barrier to adoption. Very little is known about the actual or optimal states of the eHealth workforce needs of LMICs. The objective of this study was to develop a framework to characterize and assess the eHealth workforce of hospitals in LMICs.

**Methods:**

To characterize and assess the sufficiency of the workforce, we designed this study in twofold. First, we developed a general framework to categorize the eHealth workforce at any LMIC setting. Second, we combined qualitative data, using semi-structured interviews and the Workload Indicator of Staffing Needs (WISN) to assess the sufficiency of the eHealth workforce in selected hospitals in a LMIC setting like Ghana.

**Results:**

We surveyed 76 (60%) of the eHealth staff from three hospitals in Ghana—La General Hospital, University of Ghana Hospital, and Greater Accra Regional Hospital. We identified two main eHealth cadres, technical support/information technology (IT) and health information management (HIM). While the HIM cadre presented diversity in expertise, the IT group was dominated by training in Science (42%) and Engineering (55%), and the majority (87%) had at least a bachelor’s degree. Health information clerk (32%), health information officer (25%), help desk specialist (20%), and network administrator (11%) were the most dominant roles. Based on the WISN assessment, the eHealth workforce at all the surveyed sites was insufficient. La General and University of Ghana were operating at 10% of required IT staff capacity, while Ridge was short by 42%.

**Conclusions:**

We have developed a framework to characterize and assess the eHealth workforce in LMICs. Applying it to a case study in Ghana has given us a better understanding of potential eHealth staffing needs in LMICs, while providing the quantitative basis for building the requisite human capital to drive eHealth initiatives. Educators can also use our results to explore competency gaps and refine curricula for burgeoning training programs. The findings of this study can serve as a springboard for other LMICs to assess the effects of a well-trained eHealth workforce on the return on eHealth investments.

**Electronic supplementary material:**

The online version of this article (10.1186/s12960-018-0330-8) contains supplementary material, which is available to authorized users.

## Background

Health systems in low- and middle-income countries (LMICs) have faced considerable challenges in providing high-quality, affordable, and universally accessible care. eHealth is one of the most promising solutions to address these challenges [1–4]. The World Health Organization (WHO) defines eHealth as “the cost-effective and secure use of information and communication technologies in support of the health and health-related fields including healthcare, health surveillance and health education, knowledge and research [5].” eHealth technologies can strengthen health systems by alleviating distance and time barriers via telemedicine and continuing medical education and support decision-making through clinical decision-support systems [6, 7]. In LMICs, where resources are limited, the effect of eHealth on health economics and outcomes is promising [3, 8].

Recognizing the impact that eHealth could have on healthcare delivery, particularly in LMICs with limited resources and rising populations, the WHO called on member states in LMICs, particularly in sub-Saharan Africa, to adopt and implement effective eHealth strategies to improve their health systems [9]. The Ouagadougou and Algiers declarations [10, 11], including the Frameworks for their Implementation [12, 13], initiated a movement of developing eHealth strategies across several sub-Saharan African member states, including Ghana.

In 2010, the government of Ghana, through the Ministry of Health and Ghana Health Services (GHS), rolled out an eHealth strategy framework [14], with the intent of systematically deploying systems to improve healthcare delivery and the health status of the citizenry. According to the framework, four main strategic themes were outlined. First, to streamline the regulatory framework of health data and information management. Second, to build sector capacity for the wider application of eHealth solutions within the health sector. Third, to bridge equity gaps and increase access within the health sector via the utilization of eHealth. Last, to move towards electronic records and reporting systems [14].

The commitment to embrace technology to augment healthcare delivery puts Ghana ahead of most sub-Saharan African countries [15]. Nonetheless, the potential of the country’s eHealth strategy cannot be fully realized without a well-trained workforce [14, 16]. Although capacity building is one of the four main themes of Ghana’s eHealth strategy, very little, if at all, is known, about the characteristics of the required workforce. Thus, there is a critical need to identify the requisite numbers and balanced mix of eHealth cadres, including their training needs, necessary for effective and efficient adoption of eHealth [1, 17].

We had four objectives for this work. First was to understand the characteristics of the eHealth workforce at selected health facilities in Ghana. Second was to evaluate the sufficiency of the workforce for operating and managing eHealth systems at the selected facilities. Third was to identify the nature and extent of specific gaps that could provide guidance to capacity-building and workforce development efforts. Last was to develop methods and models that might be applied in other LMICs.

## Methods

### Study design

To effectively characterize and assess the sufficiency of the eHealth workforce, we designed this study in two parts. First, we developed a framework to enable the categorization of the workforce. Second, we applied a quasi-mixed method of qualitative and quantitative data to facilitate the estimation of workload and sufficiency of a selected workforce, using the WHO’s Workload Indicator of Staffing Needs (WISN) [26].

### Framework development

Effective characterization of the eHealth workforce required a reference framework describing workforce categories, including specific job functions. However, such a framework does not exist in most LIMCs like Ghana. Therefore, there was the need to draw evidence from literature as well as to adapt models from other countries to characterize the workforce, while taking context into consideration. We developed a framework based on eHealth job definitions, roles, and competencies recommended by key organizations including Canada’s Health Informatics Association (COACH), the U.S. Office of the National Coordinator for Health Information Technology (ONC), the American Medical Informatics Association (AMIA), and the Health Information and Management Systems Society (HIMSS) [17–23].

Our framework (Table 1) represents a range of eHealth workforce roles required for effective leadership, management, support, or operations. Inspired by the work of Covvey et al. [20], including informed entries from COACH, ONC, AMIA, and HIMSS, the framework consists of three main components, namely, macro-roles, micro-roles, and functions. In a healthcare setting, each member of the eHealth workforce is considered as playing a macro-role. This role could be a leadership/management position or a support staff in one of two eHealth cadres—information technology (IT) and health information management (HIM). To address daily challenges (e.g., systems deployment, maintenance, or resource planning) in a macro position, an eHealth professional must perform specific micro-roles. These micro-roles can be formulated into designated job titles, including their specific functions or job descriptions. Examples of a micro-role for an IT designated macro-role are programmer, network administrator, or help desk specialist. Similarly, health information clerk or medical records officer are examples of micro-roles for the HIM macro-role category. We adapted this framework from Covvey et al. [20] to provide a guide for health system managers in LMICs to manage their eHealth system.

**Table 1:** eHealth cadre roles needed for leadership, management, support, or operations of eHealth systems that have been deployed in healthcare facilities based on definitions, roles, and requisite competencies from recommendations by [18–20, 22–25]

Macro-roles	Micro-roles	Functions
Leadership/management	Chief information officer	Providing high-level leadership to determine the needs of the organization based on both the current situation and the organizational strategic plan
	Director	Managing the day-to-day tactical, logistics, and functional aspects of the eHealth system
	Project manager	Directing the implementation and operation of information systems, including supervision of other involved personnel
	Project coordinator	Moderating and ensuring an effective synergy between the implementation team and end-users
	Administrator	Managing the business of healthcare including logistics, human resources, planning and finance
Technical/software support	Programmer	Creating effective data communications interfaces between systems, including (as necessary) connecting hardware and installing, modifying, and developing software
	Database administrator	Designing, maintaining, and protecting database systems
	Network administrator	Designing, maintaining, and supporting local-area and wide-area computer telecommunication networks
	Help desk specialist	Working with end-users to troubleshoot problems and questions that arise during routine use of their information system
Health information management	Health information clerk	Capturing, recording, storing, and retrieving information about a consumer and their interactions with the healthcare system
	Health information officer	Setting up, maintaining and managing systems for collection, collation, analysis, interpretation, and dissemination of health information for research, planning, and management of health services in the facility
	Analyst	Retrieving, analyzing and reporting information for direct patient care or population health
Training/quality development	Trainer	Working with end-users to educate them about the features and proper operation of their information system
	Quality assurance and improvement specialist	Working with end-users to test each component of the information system and assure that the components work effectively with each other as they are integrated. Analyzes and improve processes at every level; from care of the individual consumer through to public health and health policy

### Study setting and cohort

To understand the characteristics of the eHealth workforce, including their sufficiency, we studied selected hospitals in Ghana. For inclusion criteria, we considered location (must be situated within the Greater Accra Region), it must be easily accessible, it must have both inpatient and outpatient settings, and it must have implemented some form of an electronic health management information system. To this end, we identified three hospitals, namely, La General Hospital (La), University of Ghana Hospital (UG), and Greater Accra Regional Hospital (Ridge).

La is a municipal/district hospital in the Greater Accra Region of Ghana. It has a fully operational electronic health records (EHR) system called “CAREWEX EMR”, distributed by Queauji Consulting Ltd., Ghana. This EHR was installed in 2014 and had dedicated modules for doctors (consulting room), nurses (outpatient care), pharmacy (medication dispensing), microbiology laboratory, and billing. UG is a quasi-governmental—officially owned by the University of Ghana—hospital, which is situated within the La-Nkwantanang-Madina district of the Greater Accra Region. Like La, the UG hospital had a fully operational EHR called “HIS”, procured from IPMC Ghana. Installed in 2015, it includes modules for patient registration, outpatient-care triaging, microbiology laboratory, consulting room management, inpatient management, pharmacy, billing, and stock management. Ridge is a regional hospital for the people of Greater Accra. In the year 2016, it acquired an EHR called “HEALTH PRO ©”, developed by Spagad Technologies Limited, Ghana. At the time of our field study, the EHR was not operational. However, the vendor was running training workshops for end-users, in collaboration with the hospital’s IT team. It was expected to “GO-Live” when the hospital moves into its new facility. HEALTH PRO has dedicated modules like those found in “HIS”, in addition to a simple reporting tool.

To assess the adequacy and characteristics of the eHealth workforce for each of the study sites, we interviewed a convenience sample of the eHealth staff. We defined the eHealth workforce as all hospital employees whose work involves technical/software support (IT) and health records/information management (HIM). We invited all employees of these departments to participate in the study. The participants were on either full-time (permanent) or temporary employment contracts. Temporary staff, herein, refers to persons who work under the National Service Scheme (NSS). The NSS is a mandatory 1-year employment contract that provides graduates from tertiary institutions the opportunity to have practical work experience, either in the public or private sector. Most state institutions like the Ministries of Education and Health utilize NSS to support their personnel needs.

Table 2 provides a summary of the study setting in terms of outpatient volume, number of admissions, and inpatient bed-capacity. In addition, it provides a breakdown of the workforce in terms of the total (hospital-wide), eHealth, and study cohort.

**Table 2:** Characteristics of study setting and workforce. Bed capacity, outpatient attendance, and inpatient admission figures are all based on end of year (2016) reports

Hospital	Outpatient attendance	Inpatient admission	Beds	Workforce N (F/T)		
				General	eHealth	Sample
La	90 489	8 194	161	445 (375/70)	38 (16/22)	20 (5/15)
Ridge	111 059	16 934	620	785 (654/131)	65 (29/36)	47 (29/18)
UG	95 428	6 290	130	370 (307/63)	23 (15/8)	9 (5/4)

*N* total number, *F* full-time, *T* temporary staff (National Service Scheme)

### Data collection

To understand the characteristics of the study cohort as well as assess their workload, we devised semi-structured interviews using a questionnaire. In addition, we collected other relevant data through observations, review of human resource policies, and end of year reports at the study sites.

The questionnaire (see Additional file 1), which was administered by the lead author herein, consists of two main parts: (1) background information and (2) work tasks. The aim of the first part is to understand the characteristics of the eHealth worker. Participants were asked to provide basic information about their background such as age, gender, job title, contract type, the highest level of education, and their domain of training. The aim of the second is to facilitate the estimation of workload and sufficiency. Participants were asked to list their daily work tasks and the average time spent on each task.

To minimize potential variations in participants’ response, we observed (non-participatory) a random sample of about 45 (60%) of the participants to estimate the average time they spent on work-related activities. At the patient registration desk, for instance, the lead author observed and recorded the time the health information clerks/officers spent on registering a new patient or verifying health insurance. We transcribed all the listed daily work tasks, including the times spent on them, and categorized them according to listed job titles (see Additional file 2). This approach enabled us to average out the workload components and working times; key inputs for assessing the sufficiency of the eHealth workforce, using the WISN method.

Finally, other relevant quantitative data regarding facility capacity, staff strength, and patient volume were collected from end-of-year (2016) reports of the surveyed hospitals.

### Ethics

We obtained approval for two ethical clearances for this study. First, we acquired a local clearance, with a protocol identification number of ECBAS 023/16-17, from the Ethics Committee for Basic and Applied Sciences, University of Ghana. Second, we obtained an approval (IRB number: PRO17030491) from the Institutional Review Board of the University of Pittsburgh.

### Assessing sufficiency of the eHealth workforce

To assess the sufficiency of the eHealth workforce, we used WISN [26, 27] to estimate staff shortage/excesses and workload pressure at the study sites. WISN is a human resource planning tool that could be used to estimate the number of health workers of a cadre that is required to manage the actual workload in a given hospital. It can also be used to estimate and standardize workload and the time spent to do specific activities.

The WISN method requires a few steps. First, determine the priority cadre(s) and the type(s) of health facility. Second, estimate the available working time. Third, define workload components. Fourth, set activity standards. Fifth, establish standard workloads. Six, calculate allowance factors. Last, compute the staffing requirements based on WISN. The WISN manual describes these steps in detail [27].

The priority cadres, herein, are the two main eHealth workforces—IT and HIM; and the types of health facilities are the three surveyed sites—La, UG, and Ridge.

Available working time (AWT) for the eHealth worker is the time available in 1 year to do their work—that is number of possible working days minus absences (both authorized and unauthorized). The average AWT for a Ghanaian health worker is 205 days/year (1640 h/year) considering the number of possible working days in a year (260) and absences due to statutory public holidays (13), average sick leave (5), entitled leave of absence (25), special no notice leave (7), and mandatory training (5) [28].

Workload components are the tasks performed by an eHealth staff on a given day. There are three types of workload components: eHealth service activities (eHSAs), support activities, and additional activities. eHSAs are performed by all members of a cadre and measured through regular collection of statistics—registration of patients, for example. Support activities are also performed by all members of a cadre, but regular statistics are not collected—attending meetings, for instance. Additional activities are performed only by certain members of the cadre—data analysis, for instance, is done by a section of the HIM cadre.

An activity standard is defined as “the time necessary for a well-trained, skilled and motivated worker to perform an activity to professional standards in the local circumstances [27].” Service standard and allowance standard are the two main types of activity standards. A service standard is an activity standard for an eHSA. It can be expressed as unit time or rate of working. For example, the service standard for patient discharge by a HIM staff member can be expressed as “10 minutes per inpatient” or “25 admitted patients per inpatients day”. An allowance standard, on the other hand, is an activity standard for support and additional eHealth activities. An IT staff member, for instance, can spend “one hour per working day” on “report writing”.

We extracted the workload components, including the average time spent on each activity, from the questionnaire responses, and together with our observations, we average them out to set the activity standards. Due to a small number of available eHealth staff, there was a multiplicity of overlapping activities across the job titles. It was thus challenging to draw a distinction between activities among, for instance, a health information clerk, a medical records officer, and a health information officer. Consequently, we coalesced the activities into a generalized HIM or IT activity standard for the two main eHealth cadres, defined herewith (see Additional file 3).

The standard workload is the amount of work within an eHealth work activity that one staff can do within a year [27]. Formally, it is the AWT divided by the activity standard. If a HIM staff member, for instance, has 98 400 min per year to work, but spends 4% on meetings, her actual working time is 94 464 min per year. If she spends 10 min to register new patients at the outpatient department (OPD), then the standard patient-registration workload is 9 446 (94 464 /10) patients per year. Thus, we computed the standard workloads for all eHSA.

For expediency, we made the following decisions for estimating eHSA standard workloads. For the HIM cadre, we used annual OPD attendance to represent the annual load for registering new patients and scheduling a doctor’s appointment; the workload for admission/discharge was based on annual inpatient admissions, and the total number of insured patients were 60% of annual patient volume. For the IT cadre, we surmised that every four health workers were assigned three computing hardware components (i.e., desktop computer, laptop, or printer), and on the average, three computing software packages were installed per system. In addition, a staff with a senior role can supervise up to ten junior colleagues. These figures were culled from the hospitals’ end-of-year report and the GHS' five-year human resource plan for Ridge [29].

We leveraged the workload components and activity standards, as illustrated in the WISN manual [27], to estimate the WISN_SCORE_ for each major cadre—with and without temporary workers—at each health facility. In addition, we used the WISN_SCORE_ to evaluate two different aspects of the eHealth staffing situation at the study sites. First, we estimated staffing surplus or shortage by computing the difference between the current and required number of staff. A positive number indicates staffing surplus, while a negative figure implies shortage. Second, we estimated workload pressure by computing the ratio between current and required staff. A ratio of one suggests a balance between the current staffing levels and workload. A ratio that is greater than one is indicative of overstaffing, while a ratio of less than one suggests that the staffing levels may not be sufficient enough to cope with the workload. The smaller the ratio the higher the pressure. We set a ratio below 0.7 as high pressure, or low otherwise. Herein, the eHealth workforce is insufficient if it suffers staff shortage and high workload pressure.

## Results

### General distribution of surveyed workforce

Table 3 represents a summary of the surveyed eHealth workforce across the three locations and grouped by gender, type of contract, and eHealth cadre type. Ridge had a disproportionately higher number of the surveyed workforce due to two reasons. First, it is relatively larger in capacity and hence serves a larger patient population. Second, at the time of the survey, the hospital was in a process of moving into an ultra-modern 620-bed facility that required an increase in IT workforce, albeit temporarily, to support and manage the installation of software and hardware as well as train the end-users.

**Table 3:** A summary of the surveyed eHealth workforce

	IT				HIM				Total
	Full-time		Temporary		Full-time		Temporary		
Facility	Female	Male	Female	Male	Female	Male	Female	Male	
LA	0	1	2	8	2	2	1	4	20
Ridge	0	7	1	11	9	13	2	4	47
UG	0	1	0	0	1	3	0	4	9
Total	0	9	3	19	12	18	3	12	76

Out of the 76 staff surveyed, only 18 (24%) were female; they were even less represented in the IT category than the HIM. We recorded a total of 3, that is, 9.7% of the IT group itself and 4% of the total surveyed eHealth staff. The gender distribution improved, relatively, within the HIM group; the ratio between female and male was 1:2 (i.e., 33% female). These statistics suggest that women are starkly underrepresented in the eHealth workforce.

Similarly, 39 (51%) were full-time employees. Of this cohort, 5 (13%), 29 (74%), and 5 (13%) worked at LA, Ridge, and UG, respectively. LA and Ridge employ relatively more temporary workers, that is, 15 (41%) and 18 (49%), respectively, as opposed to 4 (11%) from UG. This is not surprising since LA and Ridge are fully owned by the government, which heavily relies on NSS to fill employment gaps. Furthermore, we observed a relatively high proportion of (about 71% as compared to 33% of HIM) temporary staff among the IT group. This apparent overreliance on temporary staff might be attributed to two possible causes; the GHS may not have enough resources to employ full-time IT staff, or there is a shortage of IT personnel within the health sector, if not the country at large.

### Domain of training and level of expertise

Table 4 shows the relationship between the domains of training and the highest level of education of the surveyed eHealth staff. Overall, the number of surveyed staff who were trained in Science, Engineering, Health Information, Management, Humanities, or Other, was respectively 24 (32%), 20 (26%), 12 (16%), 8 (11%), 7 (9.2%), or 5 (6.6%). In addition, the number of those who have attained a Bachelors’, a Certificate, a High National Diploma (HND), or a Master’s degree was 47 (62%), 20 (26%), 7 (9.2%), or 2 (2.6%), respectively. Here, Bachelor’s denote BA, BSc, or BEng degrees; Certificate referrers to a diploma from a high school, vocational institution, or a community college; and Master’s represent a graduate level degree such as MA, MSc, MBA, MEng, or MPhil.

**Table 4:** The distribution of domain of training relative to degree of expertise per eHealth cadre

Domain of training	IT				HIM				Total
	Cert.	HND	BA	MA	Cert.	HND	BA	MA	
Engineering	2	0	15	0	0	0	3	0	20
Health information	0	0	0	0	12	0	0	0	12
Humanities	0	0	1	0	1	1	4	0	7
Management	0	0	0	0	2	1	4	1	8
Other	0	0	0	0	1	1	3	0	5
Science	0	2	10	1	2	2	7	0	24
Total	2	2	26	1	18	5	21	1	76

*Cert.* Certificate, *HND* High National Diploma; *BA* Bachelor’s Degree (BA/BSc/BEng); *MA* Master’s Degree (MA/MSc/MBA/ MEng /MPhil); *IT* Information Technology, *HIM* health information management

The HIM cadre shows a diversity of skill and level of expertise. While Engineering represented the smallest subset (6.7%), Health Information had the highest (27%), followed closely by Science (24%). The domination of Health Information here is not too surprising as members of this group were trained at the Kintampo College of Health, which offers a dedicated certificate program in Health Information Management. The domain distribution within the IT cadre was not surprising; it was heavily dominated by Engineering (55%) and Science (42%). In fact, the only other represented domain—an outlier—was Humanities (3.2%). Meanwhile, the IT cohort appeared to be highly skilled; 87% of them had at least a Bachelors’ degree.

### Distribution of job titles

Table 5 displays the distribution of job titles/roles as indicated by the study participants. In addition, we show the distribution of the job roles relative to the total number of inpatient beds (bed-size) and the total patient volume (outpatient + inpatient) per hospital.

**Table 5:** The distribution of job roles relative to bed-size and patient-volume

Job Role	LA			Ridge			UG		
	# Staff	# Beds	# Patients	# Staff	# Beds	# Patients	# Staff	# Beds	# Patients
Database administrator	2	81	49 342	1	620	127 993	0	NA	NA
Health information clerk	5	32	19 737	14	44	9 142	5	26	20 344
Health information officer	3	54	32 894	14	44	9 142	2	65	50 859
Help desk specialist	2	81	49 342	12	52	10 666	1	130	101 718
Manager (HIM)	0	NA	NA	1	620	127 993	1	130	101 718
Manager (IT)	1	161	98 683	2	310	63 997	0	NA	NA
Network administrator	7	23	14 098	1	620	127 993	0	NA	NA
Programmer	0	NA	NA	1	620	127 993	0	NA	NA
Systems analyst	0	NA	NA	1	620	127 993	0	NA	NA
Total	20	8	4 934	47	13	2 723	9	14	11 302

# Staff = number of staff; # Beds = number of beds; # Patient = number of patients

Because the HIM cadres are heavily involved in the management of patients’ information at the point of care, their numbers are almost proportional to bed size and patient volume. This is reflected in the numbers for health information clerk and health information officer. Similarly, the help desk specialists and network administrators were the most prevalent within the IT group, since they were heavily involved in troubleshooting the eHealth infrastructure as well as assisting the end-users on how to navigate the newly installed systems. In summary, the results in Table 5 are arguably a general reflection of the current eHealth needs and priorities for these hospitals. While the majority of the macro-roles (see workforce framework in Table 1) were covered, medical records management and IT support (helpdesk) roles were over-represented.

### Assessing sufficiency of the eHealth workforce

Table 6 presents the steps for estimating HIM staff requirement for La, based on WISN. For brevity, we randomly select La General hospital to illustrate the procedure. Additional files 4 and 5 provide the full computations for all other cadres and hospitals.

**Table 6:** Determining the HIM staff requirement at La General Hospital, based on WISN. AWT = 1 640; OPD attendance = 90 489; inpatient admission = 8 194; number of HIM staff = 31

eHealth service activity of all HIM staff	Workload component	Service standard (minutes/case)	Annual workload	Standard workload	Required number of staff
	Registration of new patients	5	90 489	19 680	4.6
	Scheduling of appointments	5	90 489	32 800	2.8
	Admission/discharge of patients	3	8 194	19 680	0.4
	Verification of health insurance	3	59 210	32 800	1.8
	Assist patients with care pathway	2	90 489	49 200	1.8
	A. Total required staff for health service activities				11.4
Support activities of all HIM staff	Workload component	CAS (actual working time)			CAS (% working time)
	Storing and retrieving patients’ data	2 h per day			25
	Maintaining and securing health records	1 h per day			12.5
	Meetings	3 h per month			2.2
	Total CAS percentage				39.7
	B. Category allowance factor: {1 / [1 − (total CAS percentage / 100)]}				1.7
Additional activities of certain HIM staff	Workload components	No. of staff performing the work		IAS (hours per person)	Annual IAS (for all staff performing activity)
	Data analysis	3		4	2 460
	Report writing	3		4	2 460
	Technical training and supervision	3.1		2	1 271
	Processing of insurance claims	4		0.17	18 090.6
	Administrative duties	1		1	205
	Report submission	1		2	104
	Total IAS in a year				24 590.6
	C. Individual allowance factor (annual total IAS/AWT)				14.9
	Total required number of HIM staff based on WISN: (A × B + C)				33.9

The table estimates the WISN_SCORE_ separately for three different HIM-based workload groups—eHealth service activities, support activities by all HIM staff, and additional activities by certain members of the HIM cadre. Based on end of year (2016) statistics, the HIM staff registered, scheduled appointments, or assisted 90 489 patients at the OPD. They admitted 8194 inpatients and verified health insurance for 59 210. Meanwhile, the estimated standard workloads for registering or admitting a patient, scheduling appointment or verifying health insurance, and assisting patients are respectively 19 860, 32 800, and 49 200.

Thus, La requires 4.6 (90 480/19 860) HIM staff to cope with patient registration or a total of 11.4 workers (4.6 + 2.8 + 0.4 + 1.8 + 1.8) to cope with all HIM-specific eHealth service activities. Next, we multiply the eHSA by the category allowance factor (CAF) (11 × 1.7) to obtain how many HIM workers (19) La require to cope with both eHSA and support activities workload. Note that the CAF was derived from the total category allowance standard (CAS) (40%). That is, all HIM staff spends 25% of their work-time for information storage and retrieval, 13% for records maintenance and security, and 2.2% for attending work-related meetings. Finally, we add the workload (IAF = 15) due to additional activities (e.g., report writing, technical training, and supervision) that are done by only certain members of the HIM staff. Thus, La General hospital requires a total (WISN_SCORE_) of 34 (≈ 19 + 15) HIM workers to cope with all workload components.

Table 7 displays results for assessing the sufficiency of the eHealth cadres at the three study sites, based on WISN. For each cadre type at each site, we show sufficiency based on the current staffing levels of only full-time staff or a combined staff of full-time and temporary.

**Table 7:** Assessing staff sufficiency of the eHealth cadre using WISN results

Health facility	Cadre type	Staff mix	Current number	Required no., based on WISN	Shortage or excess	Workforce problem	WISN ratio	Workload pressure
LA	IT	F	1	10	−9	Shortage	0.1	High
		F + T	7	11	−4	Shortage	0.6	High
	HIM	F	15	33	−18	Shortage	0.5	High
		F + T	31	34	−3	Shortage	0.9	Low
Ridge	IT	F	7	12	−5	Shortage	0.6	High
		F + T	19	13	6	Surplus	1.5	None
	HIM	F	22	41	−19	Shortage	0.5	High
		F + T	46	42	4	Surplus	1.1	None
UG	IT	F	1	10	−9	Shortage	0.1	High
		F + T	1	10	−9	Shortage	0.1	High
	HIM	F	14	35	−21	Shortage	0.4	High
		F + T	22	35	−13	Shortage	0.6	High

*F* only full-time staff, *F + T* full-time and temporary combined

Table 7 reveals that all three hospitals experienced a shortage of IT staff when we considered only the full-time employees; these workers also experience high workload pressure. La and UG were operating with only 10% of their IT staffing requirements, while Ridge was short with about 42%. La was still short—36% below required capacity—when we combined full-time and temporary IT staff. While UG had no recorded temporary staff, Ridge had an excess (46% more than required) of IT staff. Similarly, the HIM cadres at the three hospitals are short of staff when we consider only full-time employees. That is, La, Ridge, and UG are respectively operating at about 45%, 54%, and 40% of their HIM staffing requirements. Apart from Ridge, all the other hospitals were still understaffed when we combined full-time and temporary staff. While Ridge was in excess of about 9.5% of their HIM staffing requirements, La and UG were still short by 8.8% and 37%, respectively. Note that, while a combined full-time and temporary staff of La was short of WISN requirement, the workload pressure was relatively low. In summary, the results portray an eHealth workforce that is understaffed and insufficient to cope with the workload. In addition, the hospitals need more IT personnel as compared to HIM.

## Discussion

Health systems in Ghana and most LMICs are increasingly embracing eHealth technologies into mainstream healthcare services. While these developments provide enormous potential to improve efficiency, quality, and outcomes, their success hinges on appropriately skilled workforces. Although there are wide variations across regions in the needs and availability of eHealth workforce, very little is known about the requisite mix and size of the workforce that would be needed to effectively manage an eHealth system in any given healthcare facility. In this study, we adapted a framework that could be customized and used by health systems in LMICs to categorize eHealth workforces for effective resource planning. Results from our case study in Ghana show a workforce that is characterized by inadequate numbers and an unbalanced skill mix. The imbalance within the workforce was pervasive as it cut across gender, type of contract, and expertise.

Though women were generally underrepresented in the study population, their disproportionate representation within the two main eHealth cadres may support the notion that females are represented more in clerical work (e.g., HIM) than technical work such as IT [30]. To improve diversity, it would be worthwhile to encourage more females into the eHealth workforce. Such a policy, however, would be challenging to actualize since women, in general, are underrepresented in the Science, Technology, Engineering, and Mathematics (STEM) fields.

Based on WISN requirements, there is a general paucity of eHealth staff, particularly in terms of full-time staff. Most of the temporary workforce is employed under the NSS. For decades, the majority of public institutions in Ghana, like the GHS, have relied on the NSS to fill staffing gaps. The sustainability and effectiveness of this model are highly debatable. On the one hand, the NSS is fraught with inherent challenges, including high turnover and communication costs. NSS employees are not necessarily domain experts; it takes a while to train them. Hence, the 1-year tenure may not be enough for a reasonable return on the time and resources invested in their training—particularly, the IT cadre, which is disproportionately comprised of temporary workers. On the other hand, the staffing shortage and workload pressure would have been dire without the support of NSS employees. In addition, the NSS employees cost far less and help agencies with relatively low budget an opportunity to meet their manpower needs.

The relatively low representation of full-time staff could be due to several reasons: (1) a general paucity of skilled IT staff in the country, (2) the compensation package for IT personnel may not be as attractive as other sectors of the economy, like banking or telecommunications, putting the GHS at a competitive disadvantage, and (3) because the field of health IT is fairly new the hospitals do not have well-established IT departments, as compared to the HIM workforce, which traditionally manages paper-based records. Arguably, IT is at the core of eHealth, and for LMICs, like Ghana, to make strides towards implementation of their eHealth strategies, a conscious effort must be made by all stakeholders—policymakers, educators, and GHS—to address the apparent demand.

Some relevant eHealth job categories, most notably clinical informatics, were not represented. Although informatics is a relatively new field, trained informaticians can provide services that are crucially important for smooth operation of eHealth systems. A standard occupational classification (SOC) is yet to be assigned for informaticians. The GHS, for instance, has SOC codes for health diagnosing and treating practitioners (29–1000), medical records and health information technicians (29–2070), and computer specialists (15–1000), but a standalone code or description for informaticians is non-existent. While dedicated informatics training programs have been running in the country since 2005, none of their graduates were identified among the surveyed workforce. This raises several pertinent questions. For instance, is there a disconnection between the industry and academia as regards training of the eHealth workforce? Are the training programs well-resourced and poised to meet the industrial demand? Attempting to answer these questions could provide strategic policy directions for the two main actors—GHS and the academy.

eHealth is capital intensive, so for LMICs, where resources are limited and constrained, getting the right levels and a mix of a skilled workforce is crucially important in determining the return on investment. Thus, within available resources, organizational needs, and priorities, managers of health systems must strive to assemble the most effective mix of staff. The WHO, for instance, has developed a topology of techniques that provide practical guidelines on how to address the skill mix challenges in general healthcare delivery [31], which coupled with the framework presented herein, could be equally available to develop an effective and efficient mix of eHealth workforce. While the framework categorizes the workforce, it also provides the basis to analyze the distribution and balance of skills set within the workforce at any given health facility in Ghana or other LMICs. Finally, we do not intend to generalize the findings from our case study in Ghana, but to highlight a model that other LMICs could leverage to characterize and assess their eHealth workforce.

### Limitations and future work

We focused on a few hospitals in the Greater Accra Region. While we strongly believe that the outlook will not depart markedly from other regions, a comprehensive study, which surveys the eHealth workforce from selected healthcare facilities from each of the ten regions of Ghana, may be required. For convenience, we set the minimum workload pressure to 0.7, which has not been validated by the GHS. In addition, a 30% shortage of required eHealth workers might be inadequate for most healthcare settings. Potential future work could assess the impact of the rate of shortage to eHealth systems. Policymakers could leverage the results of such study for human resource planning and optimization. Furthermore, we limited our survey to eHealth professionals who are mostly in hospitals. Future study could widen the scope to include nonclinical settings like academic research institutes, software vendors, or other areas in the digital health space. Last, we did not consider the variations in sophistication or functionality of installed EHRs at the study sites. A more robust study could devise an EHR adoption model/score and explore its relationship with workforce mix and size.

## Conclusions

In this work, we developed a framework for characterizing and assessing the eHealth workforce in LMICs. Applying this model to selected hospitals in Ghana has given us a better understanding of the eHealth staffing needs. While most LMICs are adopting eHealth strategies to improve healthcare, the requisite human capital to drive these strategies have not been well-studied. The findings of this work afford policymakers and health system administrators a quantitative basis to plan and address their eHealth staffing needs. Educators and eHealth training programs can leverage the competency gaps and imbalances to tailor their curricula to meet demand from industry. Lastly, the Ghana case study could serve as a springboard for other LMICs to effectively plan their eHealth workforce.

## Additional files

Additional file 1: A questionnaire to assess the characteristics of an eHealth worker, including their workload. (DOCX 157 kb)

Additional file 2: A workbook of daily work tasks and the times spent on them. (XLSX 24 kb)

Additional file 3: Generalized activity standards for HIM and IT cadres. (DOCX 13 kb)

Additional file 4: A workbook for estimating workload and sufficiency of the IT cadre using WISN. (XLSX 23 kb)

Additional file 5: A workbook for estimating workload and sufficiency of the HIM cadre using WISN. (XLSX 24 kb)

## Abbreviations

AMIA: American Medical Informatics Association
AWT: Available working time
CAF: Category allowance factor
CAS: Category allowance standard
COACH: Canada’s Health Informatics Association
EHR: Electronic health record
eHSA: eHealth service activity
GHS: Ghana Health Service
HIM: Health information management
HIMSS: Health Information and Management Systems Society
HND: High National Diploma
IAF: Individual allowance factor
IAS: Individual allowance standard
IRB: Institutional Review Board
IT: Information technology
LMICs: Low- and middle-income countries
NSS: National Service Scheme
ONC: The Office of the National Coordinator for Health Information Technology
OPD: Outpatient department
SOC: Standard occupational classification
STEM: Science, Technology, Engineering, and Mathematics
UG: University of Ghana
WHO: World Health Organization
WISN: Workload Indicators of Staffing Need
