# Supplementary material for: Assessing Ghana’s eHealth workforce: implications for planning and training
Source: Hum Resour Health. 2018 Nov 27;16:65. doi: 10.1186/s12960-018-0330-8 (PMC6260724; doi:10.1186/s12960-018-0330-8)
Supplement: Supplementary file 1 — A questionnaire to assess the characteristics of an eHealth worker, including their workload. (DOCX 157 kb) [file 12960_2018_330_MOESM1_ESM.docx]

SECTION -1 OF 3

Health IT workforce survey – Ghana

Form Description

1. Gender

2. Age (years)

SECTION -2 OF 3

Current Job

Description (optional)

3. What is the name of your organization?

Short-answer text

4. What best describes your job title?

5. Type of contract

6. How many hours do you work in a week?

7.  In which unit do you work?

8. List of Daily Task Job

| Daily Task | Time Spent (hrs.) | **C:** Core  **S:** Support |
| --- | --- | --- |
|  |  |  |
|  |  |  |
|  |  |  |
|  |  |  |
|  |  |  |
|  |  |  |
|  |  |  |
|  |  |  |
|  |  |  |
|  |  |  |
|  |  |  |
|  |  |  |
|  |  |  |

SECTION -3 OF 3

Training

Description (optional)

9. In what domain was your training?

10. What was your highest level of education you received in this?

11. List any other domain you have training in.

12. What was your highest level of education you received in this?
