# Supplementary material for: Assessing Ghana’s eHealth workforce: implications for planning and training
Source: Hum Resour Health. 2018 Nov 27;16:65. doi: 10.1186/s12960-018-0330-8 (PMC6260724; doi:10.1186/s12960-018-0330-8)
Supplement: Supplementary file 3 — Generalized activity standards for HIM and IT cadres. (DOCX 13 kb) [file 12960_2018_330_MOESM3_ESM.docx]

**Generalized activity standard for the HIM cadre.**

**HEALTH SERVICE/CORE ACTIVITIES**

| **Activity name** | **Service Standard** | **Unit** |
| --- | --- | --- |
| Registration of new patients | 5 | Minutes /Case |
| Admission/discharge of patients | 5 | Minutes /Case |
| Verification of health insurance | 3 | Minutes /Case |
| Scheduling of appointments | 3 | Minutes /Case |
| Assist patients with care pathway | 2 | Minutes /Case |

**SUPPORT ACTIVITIES**

| **Activity name** | **Service Standard** | **Unit** |
| --- | --- | --- |
| Storing and retrieving patients’ data | 2 | Hours /Day |
| Maintaining and securing health records | 1 | Hours /Day |
| Meetings | 3 | Hours /Month |

**ADDITIONAL ACTIVITIES**

| **Activity Name** | **No of Staff** | **Service Standard** | **Unit** |
| --- | --- | --- | --- |
| Data analysis | 3 | 4 | Hours /Day |
| Report writing | 3 | 4 | Hours /Day |
| Technical training & supervision | 2 | 3 | Hours /Day |
| Processing of insurance claims | 2 | 10 | Minutes /Case |
| Report submission | 1 | 2 | Hours /Week |
| Administrative duties | 1 | 1 | Hours /Day |

**Generalized activity standard for the IT cadre.**

**HEALTH SERVICE/CORE ACTIVITIES**

| **Activity name** | **Service Standard** | **Unit** |
| --- | --- | --- |
| Software installation | 30 | Minutes /Procedure |
| Hardware installation | 25 | Minutes /Procedure |
| Troubleshooting | 60 | Minutes /Procedure |
| Technical support for staff | 45 | Minutes /Case |

**SUPPORT ACTIVITIES**

| **Activity name** | **Service Standard** | **Unit** |
| --- | --- | --- |
| Systems checks | 2 | Hours /Day |
| Systems upgrades | 2 | Hours /Day |
| Training & workshop | 8 | Hours /Month |
| Meetings | 3 | Hours /Month |

**ADDITIONAL ACTIVITIES**

| **Activity Name** | **No of Staff** | **Service Standard** | **Unit** |
| --- | --- | --- | --- |
| Network administration | 1 | 24 | Hours /Day |
| Database administration | 1 | 8 | Hours /Day |
| Server administration | 1 | 24 | Hours /Day |
| Staff training & supervision | 2 | 2 | Hours /Day |
| Presentations and report writing | 1 | 8 | Hours /Month |
| Administrative duties | 1 | 1 | Hours /Day |
